# Question Processing and Clustering in INDOC: A Biomedical Question Answering System

**DOI:** 10.1155/2007/28576

**Published:** 2007-12-05

**Authors:** Parikshit Sondhi, Purushottam Raj, V Vinod Kumar, Ankush Mittal

**Affiliations:** 1 Department of Electronics and Computer Engineering, Indian Institute of Technology Roorkee, Roorkee 247667, India

## Abstract

The exponential growth in the volume of publications in the biomedical domain has made it impossible for an individual to keep pace with the advances. Even though evidence-based medicine has gained wide acceptance, the physicians are unable to access the relevant information in the required time, leaving most of the questions unanswered. This accentuates the need for fast and accurate biomedical question answering systems. In this paper we introduce INDOC—a biomedical question answering system based on novel ideas of indexing and extracting the answer to the questions posed. INDOC displays the results in clusters to help the user arrive the most relevant set of documents quickly. Evaluation was done against the standard OHSUMED test collection. Our system achieves high accuracy and minimizes user effort.

## 

[1,2,3,4,5,6,7,8,9,10,11,12,13,14,15,16,17,18,19,20,21,22,23,24]
